# Effects of In-Season Velocity-Based Resistance Training on Boxing-Specific Performance in Collegiate Boxers: A Companion Report from a Randomized Controlled Trial

**DOI:** 10.3390/jfmk11030310

**Published:** 2026-08-09

**Authors:** Yemin Han, Yiqing Xie, Zhen Zhang, Amador García-Ramos

**Affiliations:** 1 School of Physical Education, Shanghai University of Sport, Shanghai 200438, China; 2 School of Sport, Exercise and Health Sciences, Loughborough University, Loughborough LE11 3TU, UK; 3 Department of Physical Education and Sport, Faculty of Sport Sciences, University of Granada, 18071 Granada, Spain; 4 Department of Sports Sciences and Physical Conditioning, Universidad Católica de la Santísima Concepción, Concepción 4090541, Chile

**Keywords:** boxing, punching performance, resistance training, velocity loss, randomized controlled trial

## Abstract

**Objective:** This article reports the prespecified boxing-specific component of a randomized controlled trial comparing velocity-based resistance training (VBT) with percentage-based training (PBT) in collegiate boxers. Boxing-specific performance was specified as a parallel primary outcome domain in the original ethics-approved protocol, although no single boxing-specific measure was designated as the sole primary endpoint; the five measures are therefore reported as prespecified exploratory outcomes. **Methods:** The trial was designed before participant recruitment to address two parallel research objectives: changes in general lower-limb performance and changes in boxing-specific performance. Twenty-eight male collegiate boxers were randomly allocated to VBT (*n* = 14) or PBT (*n* = 14). Results for lower-limb strength, jumping, and sprinting from the same trial have been reported previously. The present report focuses on lead and rear straight-punch (LSP and RSP) force and continuous-punch (CP) frequency over 10 s, 30 s, and 1 min. Both groups were prescribed the back squat, Bulgarian split-squat, and deadlift twice weekly for eight weeks. PBT participants completed four sets of five repetitions at 70% of one-repetition maximum (1RM), whereas VBT participants were prescribed four sets using the velocity associated with 70% 1RM and ended each set at 10% velocity loss. **Results:** The between-group differences in mean change, calculated as VBT minus PBT, were 6.97 kg for lead straight-punch force (95% CI: 0.40 to 13.54), 7.21 kg for rear straight-punch force (95% CI: 3.56 to 10.86), 4.47 punches·10 s^−1^ for 10 s continuous-punch frequency (95% CI: 0.33 to 8.62), 5.05 punches·30 s^−1^ for 30 s continuous-punch frequency (95% CI: 0.14 to 9.96), and −2.65 punches·min^−1^ for 1 min continuous-punch frequency (95% CI: −13.97 to 8.67). **Conclusions:** Greater mean improvements in straight-punch force and short-duration continuous-punch performance were observed in the VBT group, although the precision of several estimates was limited and the findings should be interpreted cautiously because multiple exploratory outcomes were analyzed without formal multiplicity adjustment. For the 1 min test, there was no statistically conclusive evidence of a between-group difference. Registration: The trial was retrospectively registered with the Chinese Clinical Trial Registry (ChiCTR2500111377), and the study materials were subsequently registered on the Open Science Framework (OSF; DOI: 10.17605/OSF.IO/VY6TS).

## 1. Introduction

Successful boxing performance depends not only on technical-tactical proficiency but also on the capacity to generate force rapidly and repeatedly during offensive and defensive actions [1,2,3,4,5,6]. Resistance training (RT) is therefore commonly incorporated into boxing preparation to develop strength, power, speed, and fatigue resistance [7]. However, improvements in general physical qualities do not necessarily transfer directly to sport-specific actions. From an applied perspective, measures such as straight-punch force and the ability to sustain rapid punching combinations may provide a more direct indication of whether an RT intervention enhances boxing performance.

Conventional percentage-based training (PBT) prescribes resistance according to a previously determined one-repetition maximum (1RM) and typically requires athletes to complete a fixed number of repetitions per set [7,8]. Although this approach is straightforward, a predetermined prescription may not account for daily variations in readiness associated with sleep, nutrition, accumulated fatigue, or psychological stress [9]. Moreover, athletes with different repetition capacities may experience substantially different levels of fatigue despite completing the same number of repetitions [10,11]. Velocity-based resistance training (VBT) attempts to address these limitations by using lifting velocity to estimate relative loading [12] and velocity loss (VL) to regulate the number of repetitions performed within each set [13]. Immediate velocity feedback may also reinforce maximal movement intent and facilitate high-velocity force production [2,14]. These characteristics may be especially relevant during the competitive season, when physical development must be balanced with technical-tactical training, recovery, and fatigue management [15,16].

Research involving soccer, rugby, basketball, and other athletic populations suggests that VBT can enhance strength- and power-related performance and, in some circumstances, may produce greater improvements than fixed percentage-based prescriptions [17,18,19]. The adaptations elicited by VBT also appear to depend on the selected VL threshold. Lower thresholds generally restrict fatigue and preserve movement velocity, whereas higher thresholds increase the number of repetitions performed and may provide a greater hypertrophic stimulus [20,21,22,23]. Nevertheless, most previous interventions have assessed general performance using outcomes such as maximal strength, jumping, and sprinting. Evidence regarding whether these adaptations transfer to sport-specific performance remains limited, particularly in combat sports.

The overall randomized trial was designed before participant recruitment to address two parallel research objectives: to compare the effects of VBT and PBT on general lower-limb performance and to determine their effects on boxing-specific performance. Findings concerning lower-limb maximal strength, jumping, and sprinting have been reported previously [24]. The present companion report focuses on the prespecified boxing-specific component of the trial by examining straight-punch force and continuous-punch frequency. Based on the importance of rapid lower-limb force transfer during punching [2,25], we hypothesized that, compared with PBT, an eight-week VBT program using a 10% VL threshold would produce greater improvements in these boxing-specific measures, particularly during shorter-duration efforts.

## 2. Materials and Methods

### 2.1. Participants and Trial Context

The overall trial was prospectively designed to investigate two parallel research components using the same randomized cohort and training intervention: general lower-limb performance and boxing-specific performance. Findings from the general lower-limb component were reported previously by Han et al. [24], whereas the present manuscript reports the prespecified boxing-specific component. The sample size was determined a priori using G*Power 3.1 (Heinrich Heine University Düsseldorf, Düsseldorf, Germany) [26] for the group-by-time interaction in a two-group, two-measurement repeated-measures design, assuming *f* = 0.30, α = 0.05, 80% power, a pre–post correlation of 0.50, and a nonsphericity correction of 1.0. Because no directly comparable boxing-specific VBT data were available, *f* = 0.30 was selected as a moderate a priori assumption. The calculation applied to the overall trial rather than to a single boxing-specific outcome and yielded a minimum sample size of 24 participants. Participants were recruited from Shanghai University of Sport between 10 and 20 February 2025. Before enrolment, all athletes received information about the study procedures, potential benefits, and risks and provided written informed consent.

An independent researcher who was not involved in participant recruitment, intervention delivery, or outcome assessment generated the allocation sequence using a computer-generated randomization procedure. The same researcher retained the allocation sequence and disclosed the group assignments only after completion of the baseline assessments. Participants were enrolled by a member of the research team who did not have access to the allocation sequence. Twenty-eight athletes were assessed for eligibility, and all met the eligibility criteria and were enrolled. They were randomly allocated to the VBT or PBT group (*n* = 14 per group). No stratification or block randomization was used. All randomized participants received the allocated intervention, completed the post-intervention assessments, and were included in the analyses in their originally assigned groups, consistent with the intention-to-treat principle. No participants withdrew or were lost to follow-up, and no outcome data were missing. No protocol deviation requiring participant exclusion or modification of the planned analyses was recorded.

Ethical approval was granted by the Scientific Research Ethics Committee of Shanghai University of Sport (No. 102772025RT044), and the trial was conducted in accordance with the Declaration of Helsinki. At enrolment, participants reported no health conditions that restricted training. They also reported no use of medication, ergogenic aids, or dietary supplements during the intervention. The baseline characteristics of the participants are presented in Table 1.

### 2.2. Trial Registration and Prespecification

The trial was retrospectively registered with the Chinese Clinical Trial Registry (ChiCTR2500111377), and the study materials were subsequently registered on the Open Science Framework (OSF; DOI: 10.17605/OSF.IO/VY6TS). Public registration occurred after participant recruitment and data collection. However, the dated ethics-approved protocol, finalized before recruitment, specified two parallel primary outcome domains without a primary–secondary hierarchy: general lower-limb performance and boxing-specific performance. The boxing-specific domain comprised lead- and rear-hand straight-punch force and continuous-punch frequency over 10 s, 30 s, and 1 min. No single boxing-specific measure was designated as the sole primary endpoint. The original ethics-approved protocol is publicly available through the OSF record and is provided as Supplementary File S1.

### 2.3. Study Design and Outcome Schedule

All assessments and training sessions took place at the Strength and Conditioning Research Center in Shanghai, China. Before the intervention, testing was distributed across three days: maximal-strength assessments were completed on day 1, individual load-velocity relationships were established on day 2, and boxing-specific outcomes were assessed on day 3. The same schedule was repeated after the intervention. Examiners responsible for outcome collection were unaware of group allocation. Maximal-strength, countermovement-jump, standing-long-jump, and 30 m sprint findings were presented in the report of the general lower-limb component [24]. The present article reports the distinct boxing-specific component, comprising straight-punch force and continuous-punch frequency. An eight-week intervention was used because periods of approximately 6–8 weeks are commonly deemed sufficient to detect initial adaptations to RT [27,28].

### 2.4. Outcome and Training Procedures

#### 2.4.1. One-Repetition Maximum Assessment

One-repetition maximum was assessed for the back squat, Bulgarian split-squat, and deadlift. Testing began with an external load of 20 kg, which was increased in 10 kg increments until mean velocity decreased below 0.5 m·s^−1^. Thereafter, load increments of 1–5 kg were used to determine the greatest load that could be lifted through the required range of motion with correct technique. Participants performed three to four repetitions when mean velocity was greater than 0.7 m·s^−1^, two repetitions when mean velocity was between 0.5 and 0.7 m·s^−1^, and one repetition when mean velocity was below 0.5 m·s^−1^. Repetitions at the same load were separated by 10 s, and successive loads by 5 min.

During the back squat, participants descended until the thighs were approximately parallel to the floor. For the Bulgarian split-squat, the rear foot was elevated on a bench and the participant descended until the front thigh was approximately parallel to the floor; the dominant and non-dominant legs were assessed separately. During the deadlift, participants used a shoulder-width stance and mixed grip and completed the lift through coordinated knee and hip extension. All attempts were supervised to verify technique and range of motion.

#### 2.4.2. Individual Load-Velocity Profiling

Individual load–velocity profiles were established separately for the back squat, deadlift, and each leg of the Bulgarian split-squat. Mean velocity (MV) was recorded using a GymAware Power Tool linear position transducer (Kinetic Performance Technologies, Canberra, Australia). After a standardized warm-up, participants completed three repetitions at 40% and 60% of baseline 1RM, two repetitions at 80%, and one repetition at 90%. GymAware has demonstrated acceptable reliability across this loading range [29]. Each concentric action was preceded by a 3–4 s pause in the lowest position to minimize assistance from the stretch–shortening cycle, and participants were instructed to lift with maximal intended velocity. The two legs were assessed in a randomized order during the Bulgarian split-squat. Repetitions at the same load were separated by 10 s, and successive loads were separated by 3 min. The fastest repetition at each load was retained. For each exercise or leg, an individual linear regression equation of the form MV = β_0_ + β_1_ × load was fitted to model the relationship between external load and MV. The velocity corresponding to 70% 1RM was derived from this individual equation and used as the target velocity for VBT. The original protocol did not specify a minimum coefficient-of-determination threshold or a formal procedure for excluding or reconstructing an inadequate profile; therefore, no retrospective fit criterion was introduced.

#### 2.4.3. Boxing-Specific Performance Tests

Boxing-specific performance was assessed using a punching bag connected to the Xingxun Boxing Training Monitoring System (NY-BX101, version 2.0; Xingxun, Shanghai, China). The NY-BX101 software (version 2.0; Xingxun, Shanghai, China) displayed punch-force values under the label “kg”. Because it could not be confirmed whether this label represented kilogram-force or another device-specific measurement scale, the outcomes are reported as manufacturer-reported device values labelled “kg”, rather than as verified SI force measurements. Before baseline testing, all participants completed a familiarization session using the same equipment and testing procedures. For each participant, the pre- and post-intervention assessments were conducted at approximately the same time of day under the same pre-test instructions regarding nutrition, hydration, sleep, and avoidance of strenuous exercise. Participants adopted their habitual stance and selected a comfortable distance from the target. For each punch, they were instructed to initiate force through the lower limbs, rotate the trunk, and complete the action with arm extension. Lead-hand and rear-hand straight-punch performance was tested first. Five valid attempts were performed on each side, with 30 s between attempts, and the highest device-reported value was retained for analysis. Continuous-punch frequency was then assessed in the fixed order of 10 s, 30 s, and 1 min to minimize the influence of fatigue from the longer-duration tests on the shorter, more explosive tests. Each duration was performed three times, with 30 s, 2 min, and 3 min of recovery, respectively, and the highest number of valid punches was retained for analysis. The same investigators supervised all trials and verified technique and valid target contact. Although previous studies using other force-based punching systems have reported high within-day and between-day reliability (ICC = 0.89–0.99) [30], these estimates are not specific to the NY-BX101 system and cannot be assumed to apply to it. Using the same device and standardized protocol at both assessments supported procedural consistency but did not establish device-specific test–retest reliability, responsiveness, or whether the observed changes exceeded measurement error.

#### 2.4.4. Resistance-Training Intervention

The resistance-training intervention was common to both prespecified research components and has been described in detail in the report of the general lower-limb component [24]. Briefly, participants were scheduled to complete two lower-body RT sessions per week for eight weeks while continuing the same technical–tactical programme. Each session included the back squat, Bulgarian split-squat, and deadlift. Both groups performed four sets per exercise and rested for 3 min between sets. In PBT, the load was fixed at 70% 1RM and each set comprised five repetitions. In VBT, the initial load corresponded to the velocity associated with 70% 1RM (accepted deviation, ±0.03 m·s^−1^), and each set was prescribed to end when MV declined by 10% relative to its fastest repetition. At each training session, the external load was adjusted according to the real-time MV displayed by GymAware to maintain the individualized target velocity within ±0.03 m·s^−1^. The baseline load–velocity profiles were not formally reassessed during the intervention; instead, the training load was autoregulated on a session-by-session basis using real-time velocity feedback. Velocity was monitored using GymAware, a device with acceptable reliability and field validity [29,31], and 1RM was reassessed after week 4 to update the PBT loads for the second half of the programme. All sessions included a 10–15 min general and boxing-specific warm-up, 3–5 min of recovery before lifting, and a supervised cool-down.

**Figure 1:**
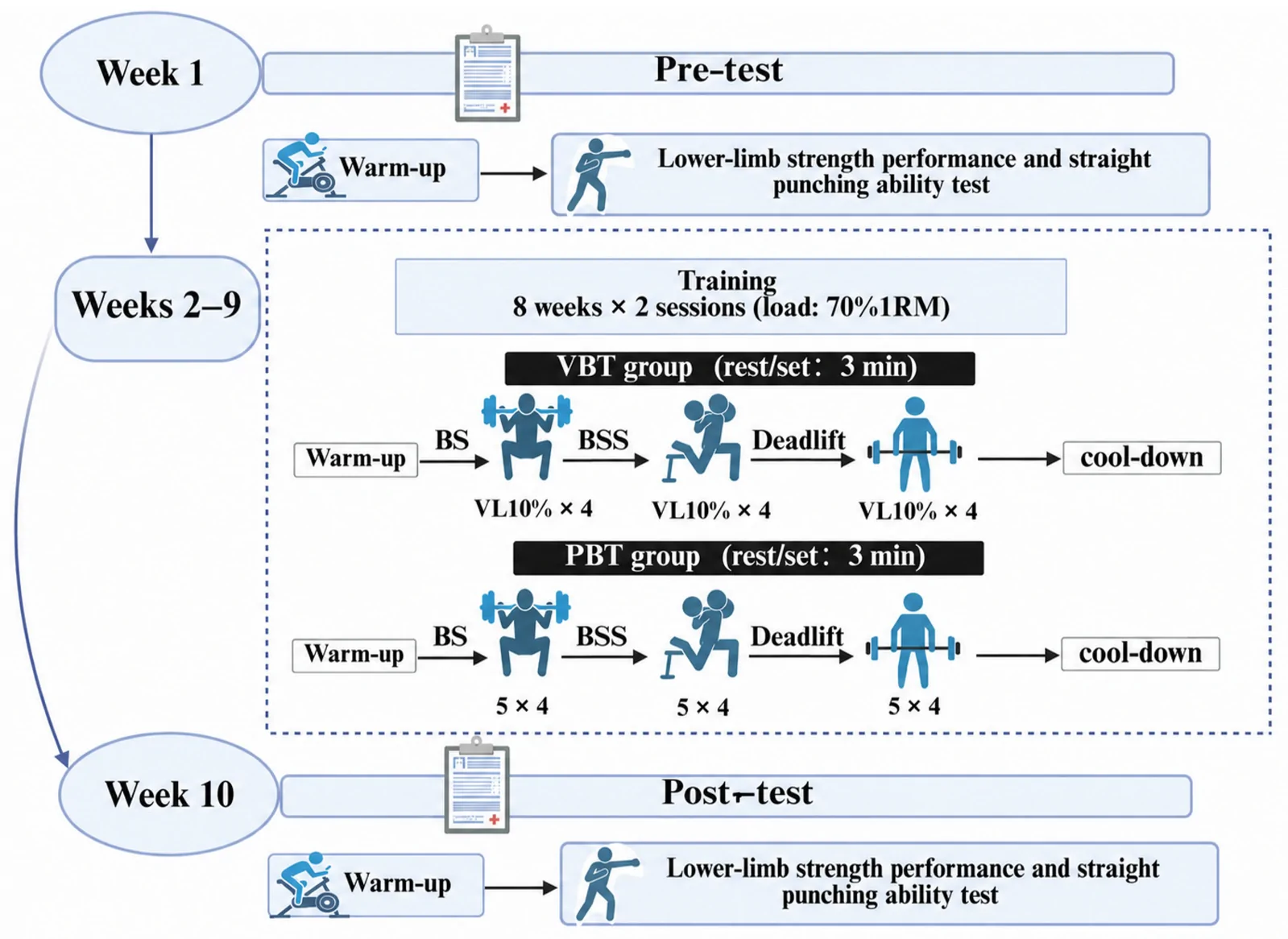
Overview of the experimental design. 1RM, one-repetition maximum; BS, back squat; BSS, Bulgarian split-squat; PBT, percentage-based training; VBT, velocity-based resistance training.

The intervention comprised 16 scheduled resistance-training sessions over eight weeks. All participants remained in the study throughout the intervention period and completed the post-intervention assessments. No training-related adverse event requiring medical attention or modification of the intervention was recorded. Session-level attendance, repetitions, external training volume, mean velocity, and achieved velocity loss were not retained in a sufficiently standardized form to support reliable quantitative fidelity analyses. Therefore, completion of all 16 scheduled sessions, adherence to the intended 10% velocity-loss threshold, and the actual VBT dose could not be quantitatively verified. An overview of the experimental design is presented in Figure 1.

### 2.5. Statistical Analysis

All analyses were conducted using SPSS 27.0 (IBM, Armonk, NY, USA). Data are presented as mean ± SD. Normality and homogeneity of variance were assessed using the Shapiro–Wilk and Levene’s tests, respectively. Separate 2 × 2 mixed ANOVAs were conducted for each boxing-specific outcome, with time (pre vs. post) as the within-participant factor and group (VBT vs. PBT) as the between-participant factor. Individual change scores were calculated as post-intervention minus baseline values. Treatment effects were expressed as between-group differences in mean change (VBT minus PBT) with 95% confidence intervals, and the corresponding *p* values were obtained from the group-by-time interaction tests.

Changes were also expressed as Hedges’ g with approximate 95% confidence intervals. Within-group effects were calculated as the pre-to-post mean change divided by the pooled pre- and post-intervention SD, whereas between-group effects were calculated as the difference in mean change between VBT and PBT divided by the pooled baseline SD. A small-sample correction was applied using $J=1-3/\left(4df-1\right)$. The normal-approximation standard error was calculated as $SE\left(g\right)=\sqrt{\left(n_{1}+n_{2}\right)/\left(n_{1}n_{2}\right)+g^{2}/\left[2\left(n_{1}+n_{2}-2\right)\right]}$, and the approximate 95% confidence interval as $g\pm 1.96\times SE\left(g\right)$. For between-group effects, $n_{1}$ and $n_{2}$ represented the VBT and PBT sample sizes and $df=n_{1}+n_{2}-2$; for within-group effects, they represented the pre- and post-intervention sample sizes within the relevant group. Because this variance procedure did not incorporate the paired pre-to-post covariance, the resulting intervals should be interpreted as approximate descriptive intervals rather than covariance-adjusted inferential intervals. Inferential interpretation therefore prioritized the mixed-ANOVA group-by-time interaction and the unstandardized between-group differences in mean change with their 95% confidence intervals. Effect sizes were interpreted as trivial (<0.20), small (0.20–0.59), moderate (0.60–1.19), large (1.20–2.00), or extremely large (>2.00) [32]. Statistical significance was set at *p* ≤ 0.05.

## 3. Results

Baseline values did not differ significantly between groups for any boxing-specific outcome (all *p* > 0.05). Significant time effects were observed for LSP force, RSP force, 10 s CP frequency, and 30 s CP frequency (*F* ≥ 5.8, *p* ≤ 0.023), but not for 1 min CP frequency (*F* = 1.2, *p* = 0.293). At the unadjusted 0.05 level, group × time interactions were observed for LSP force (*p* = 0.039), RSP force (*p* < 0.001), 10 s CP frequency (*p* = 0.032), and 30 s CP frequency (*p* = 0.043), whereas the interaction for 1 min CP frequency was not significant (*p* = 0.636) (Table 2).

The mean pre-to-post changes in the VBT and PBT groups were 8.57 and 1.60 kg for LSP force, 9.57 and 2.36 kg for RSP force, 4.71 and 0.24 punches·10 s^−1^ for 10 s CP frequency, 8.07 and 3.02 punches·30 s^−1^ for 30 s CP frequency, and 1.64 and 4.29 punches·min^−1^ for 1 min CP frequency, respectively. The corresponding unstandardized between-group differences in mean change (VBT minus PBT) were 6.97 kg for LSP force (95% CI: 0.40 to 13.54; *p* = 0.039), 7.21 kg for RSP force (95% CI: 3.56 to 10.86; *p* < 0.001), 4.47 punches·10 s^−1^ for 10 s CP frequency (95% CI: 0.33 to 8.62; *p* = 0.032), 5.05 punches·30 s^−1^ for 30 s CP frequency (95% CI: 0.14 to 9.96; *p* = 0.043), and −2.65 punches·min^−1^ for 1 min CP frequency (95% CI: −13.97 to 8.67; *p* = 0.636).

**Figure 2:**
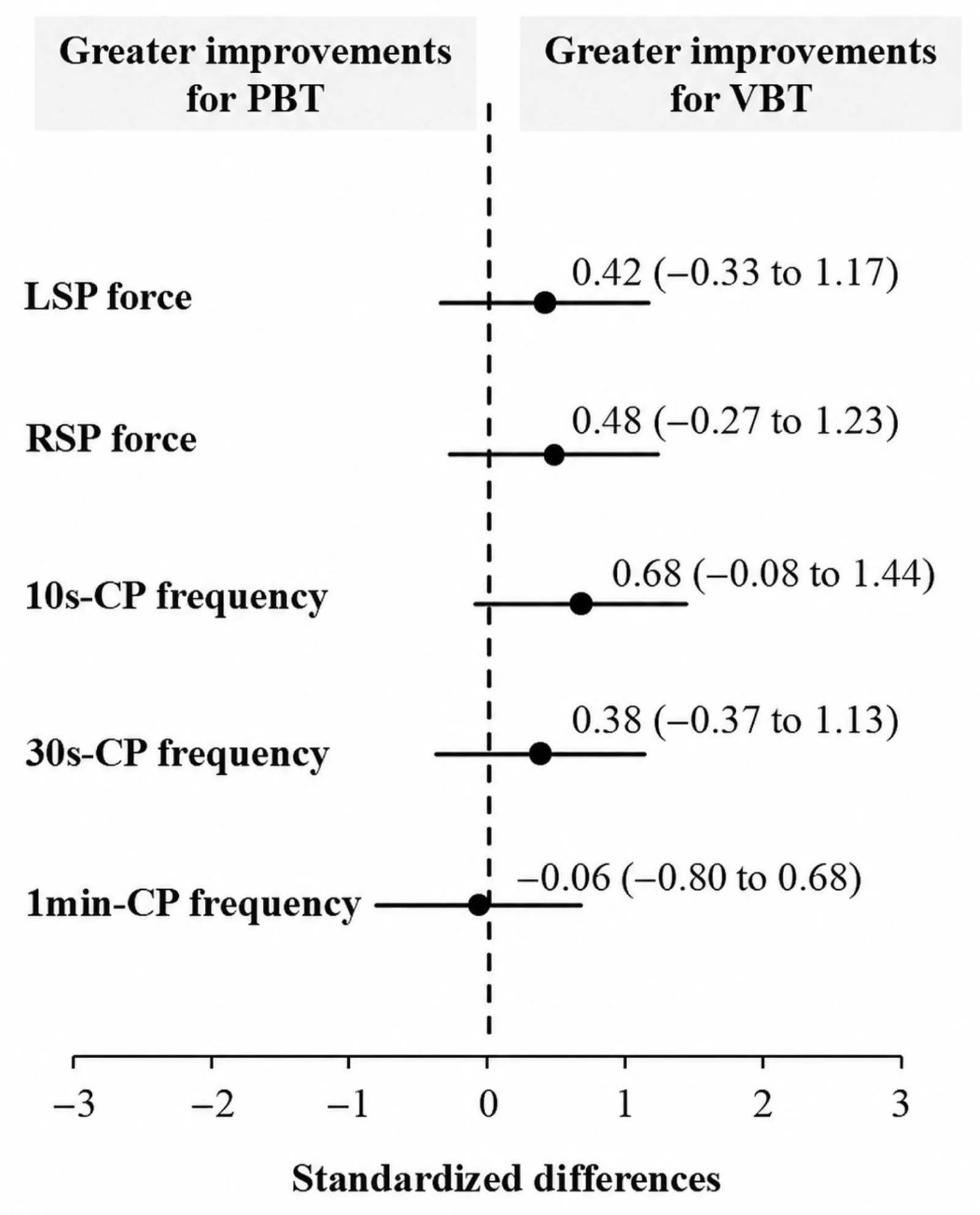
Between-group Hedges’ g values for pre-to-post intervention changes, with approximate 95% confidence intervals. Hedges’ g was calculated as the difference in mean change between VBT and PBT divided by the pooled baseline SD, with a small-sample correction applied. The confidence intervals were calculated using the normal approximation and did not incorporate the within-participant pre-to-post covariance. They should therefore be interpreted as approximate descriptive intervals and not as the confidence-interval counterparts of the mixed-ANOVA interaction tests. Positive values favor VBT, whereas negative values favor PBT. LSP, lead straight punch; RSP, rear straight punch; CP, continuous punch; PBT, percentage-based training; VBT, velocity-based resistance training.

Standardized between-group differences in change favored VBT for LSP force (*g* = 0.42), RSP force (*g* = 0.48), 10 s CP frequency (*g* = 0.68), and 30 s CP frequency (*g* = 0.38), whereas the between-group effect for 1 min CP frequency was trivial (*g* = −0.06) (Figure 2).

## 4. Discussion

The present report addresses the prespecified boxing-specific component of a larger randomized trial, whereas findings from the parallel general lower-limb performance component have been reported previously [24]. Both reports are based on the same randomized cohort and eight-week training intervention. Greater mean improvements in lead- and rear-hand straight-punch force and in the number of punches completed during the 10 s and 30 s tests were observed in the VBT group compared to the PBT group. However, the precision of several estimates was limited, and these findings should be interpreted cautiously given the modest sample size and the absence of formal adjustment for multiple outcomes. For the 1 min test, there was no statistically conclusive evidence of a between-group difference; this result should not be interpreted as evidence of equivalence. Because detailed session-level repetition and external training-volume data were unavailable for quantitative fidelity analysis, between-group differences in the delivered training exposure cannot be excluded. Rather, the findings are consistent with the possibility that regulating sets by movement velocity may be particularly relevant when performance requires rapid and forceful actions. As test duration increased to 1 min, the contribution of local upper-limb endurance and pacing may have become greater, potentially reducing the influence of the lower-body power qualities targeted by the intervention.

The present results are consistent with the lower-limb outcomes reported from the same trial [24], in which VBT produced larger improvements in countermovement jump height (*g* = 0.76), standing long jump distance (*g* = 0.76), and 30 m sprint time (*g* = 0.59). Punching is a whole-body action: force is initiated against the ground, transmitted through the lower limbs and trunk, and ultimately delivered through the upper limb [25,33,34,35,36]. Accordingly, greater lower-body explosive capacity may support both the magnitude and the rate of force transferred to the target. Correlational evidence also links jump performance with straight-punch force and punching frequency [2,37]. The parallel improvements in general explosive tests and short-duration punching outcomes therefore provide a plausible indication of transfer, although the current design cannot establish whether individual changes in lower-limb performance mediated the changes in punching performance.

Several features of the VBT prescription could have contributed to this transfer. Selecting loads from an individual load-velocity relationship allows session loads to better reflect current performance capacity than a static percentage of a historical 1RM [38,39]. Terminating sets at a low VL threshold may preserve repetition quality and limit unnecessary fatigue [13,22,28,40,41,42,43], which is particularly relevant for boxers completing concurrent technical and conditioning work. In addition, repetition-by-repetition feedback can increase movement intent, motivation, and attentional focus [44,45], and repeated exposure to feedback has been associated with favorable training adaptations [46]. These mechanisms are interdependent in the present protocol; therefore, the results support the VBT package as implemented rather than any single component in isolation.

Several limitations should be considered. First, the sample was modest and was recruited from a single collegiate training centre, which restricts generalisability to other competitive levels, female boxers, and different coaching environments. Second, no formal adjustment for multiple comparisons was applied across the five boxing-specific outcomes; the outcome-specific analyses should therefore be regarded as exploratory, particularly where interaction *p* values were close to 0.05. Third, the VBT intervention combined load autoregulation, velocity-loss-based set termination, repetition-quality control, and real-time feedback. These components were not experimentally isolated, and the findings therefore support the VBT package as implemented rather than any individual mechanism. Fourth, detailed session-level attendance, repetition, external training volume, mean velocity, and achieved velocity-loss data were unavailable for quantitative fidelity analysis. Consequently, completion of all 16 scheduled sessions, adherence to the intended 10% velocity-loss threshold, and the actual VBT dose could not be verified retrospectively. Fifth, no mediation analysis was conducted, and the proposed transfer from improvements in lower-limb explosive performance to punching performance remains hypothetical rather than causal. Sixth, device-specific criterion validity, calibration, test–retest reliability, responsiveness, and measurement-error estimates were not available for the NY-BX101 system; consequently, the absolute accuracy of the manufacturer-reported punch-force values cannot be established. Although the same device and standardized procedures were used at both assessments, this procedural consistency does not establish device-specific reliability or responsiveness, and it cannot be determined whether the observed pre-to-post changes exceeded measurement error. Seventh, the boxing tests were completed in a fixed order, and the selected recovery intervals may not have completely eliminated residual fatigue before the longer-duration assessments. Eighth, the approximate confidence intervals around Hedges’ g did not incorporate the paired pre-to-post covariance and should therefore be interpreted as approximate descriptive intervals rather than covariance-adjusted inferential intervals. Finally, concurrent technical–tactical training, sparring, conditioning, competition exposure, additional physical activity, nutrition, sleep, and recovery practices were not prospectively quantified. Unmeasured differences in these co-interventions and in the delivered resistance-training dose may therefore have influenced the observed outcomes.

## 5. Conclusions

In the prespecified boxing-specific component of this randomized controlled trial, greater mean improvements in lead- and rear-hand straight-punch force and in 10 s and 30 s continuous-punch frequency were observed in the VBT group compared to the PBT group. However, the precision of several estimates was limited, and the findings should be interpreted cautiously in view of the modest sample size and the absence of formal adjustment for multiple outcomes. For 1 min continuous punching, the data did not provide statistically conclusive evidence of a between-group difference; this finding should not be interpreted as evidence of equivalence. Considered alongside the separately reported general lower-limb component [24], the results suggest that improvements in explosive physical performance may be accompanied by benefits in short-duration boxing-specific actions. Confirmation in an independent cohort is required.

## 6. Practical Applications

In this small sample of male collegiate boxers, the observed findings suggest that an integrated VBT programme combining velocity-guided load autoregulation, velocity-loss-based set termination, and real-time feedback warrants further investigation for short-duration punching performance. These exploratory results should not be interpreted as establishing VBT as a superior or routinely recommended training strategy. Replication in larger and more diverse samples, using multiplicity-controlled analyses and validated boxing-specific measurement procedures, is required before specific implementation recommendations can be made.

## Figures and Tables

**Table 1:** Baseline characteristics of the randomized cohort. These data were reported previously in [24] and are presented here to describe the study participants.

Variable	VBT (*n* = 14)	PBT (*n* = 14)
Age (years)	19.6 ± 1.0	19.9 ± 1.0
Height (cm)	181.9 ± 7.4	179.1 ± 7.3
Body mass (kg)	77.9 ± 9.1	78.4 ± 9.5
Boxing experience (years)	6.3 ± 1.4	6.0 ± 1.5

Abbreviations: VBT, velocity-based resistance training; PBT, percentage-based training.

**Table 2:** Pre- to post-intervention changes in boxing-specific outcomes and mixed-ANOVA results for the PBT and VBT groups.

Variable/Group	Pre-Test, Mean (SD)	Post-Test, Mean (SD)	Hedges g, ES (95% CI)	ANOVA	
				Time	Interaction
lead straight punch force, kg					
PBT	54.6 (15.7)	56.2 (13.7)	0.11 (−0.64, 0.85)	*F* (1, 26) = 10.3	*F* (1, 26) = 4.7
VBT	50.3 (16.8)	58.9 (14.1) *	0.54 (−0.22, 1.29)	*p* = 0.003	*p* = 0.039
rear straight punch force, kg					
PBT	70.4 (6.8)	72.7 (7.7)	0.31 (−0.44, 1.05)	*F* (1, 26) = 44.4	*F* (1, 26) = 16.3
VBT	68.1 (19.3)	77.6 (19.8) *	0.47 (−0.28, 1.22)	*p* < 0.001	*p* < 0.001
10-s continuous punch frequency, punches·10 s^−1^					
PBT	34.9 (6.9)	35.0 (4.8)	0.02 (−0.72, 0.76)	*F* (1, 26) = 5.8	*F* (1, 26) = 5.1
VBT	34.2 (6.2)	38.9 (6.7) *	0.71 (−0.06, 1.47)	*p* = 0.023	*p* = 0.032
30-s continuous punch frequency, punches·30 s^−1^					
PBT	100.9 (12.3)	103.9 (9.7)	0.26 (−0.48, 1.01)	*F* (1, 26) = 21.7	*F* (1, 26) = 4.5
VBT	101.5 (13.6)	109.6 (14.1) *	0.57 (−0.19, 1.32)	*p* < 0.001	*p* = 0.043
1-min continuous punch frequency, punches·min^−1^					
PBT	185.5 (38.9)	189.8 (35.7)	0.11 (−0.63, 0.85)	*F* (1, 26) = 1.2	*F* (1, 26) = 0.2
VBT	183.4 (48.1)	185.1 (41.5)	0.22 (−0.70, 0.78)	*p* = 0.293	*p* = 0.636

Abbreviations: ANOVA, analysis of variance; PBT, percentage-based training; VBT, velocity-based resistance training; ES, effect size. Within-group Hedges’ g was calculated as the pre-to-post mean change divided by the pooled pre- and post-intervention SD, with a small-sample correction applied. The approximate 95% confidence intervals were calculated using the normal approximation and did not incorporate the paired pre-to-post covariance; they should therefore be interpreted as approximate descriptive intervals rather than covariance-adjusted inferential intervals. * Significant within-group difference from pre-intervention (*p* < 0.05).

## Data Availability

The original contributions presented in this study are included in the Supplementary Material. Further inquiries can be directed to the corresponding author.

## Supplementary Materials

The following supporting information can be downloaded at: https://www.mdpi.com/article/10.3390/jfmk11030310/s1, Supplementary File S1: Ethics Review Form approved by the Scientific Research Ethics Committee of Shanghai University of Sport; Supplementary File S2: Blank Informed Consent Form; Supplementary File S3: Raw data for lead and rear straight-punch force and continuous-punch frequency over 10 s, 30 s, and 1 min.
