# Supplementary material for: Effects of In-Season Velocity-Based Resistance Training on Boxing-Specific Performance in Collegiate Boxers: A Companion Report from a Randomized Controlled Trial
Source: J Funct Morphol Kinesiol. 2026 Aug 9;11(3):310. doi: 10.3390/jfmk11030310 (PMC13510493; doi:10.3390/jfmk11030310)
Supplement: Supplementary file 1 [file jfmk-11-00310-s001.zip › File S1. Ethics Review Form.pdf]

Scientific Research Ethics Committee of Shanghai University of Sport

Application Form for Ethical Review of Human Experiments

IRB: 102772025RT044 **Date:** 2025.1.20

After completing the form, please send it in word format to the email address of the Quarterly Ethics Committee at lunli@sus edu.cn. Please scan the signature page and submit it in PDF format. If you have any questions during the filling process, please send an email to lunli@sus.edu.c. Please do not modify or delete this table without authorization, please keep all the instructions in this form. Please follow the guidelines of the Human Experiment Ethics Committee for filling out the specifications.			
**Research Title**	The effect of velocity-based resistance training (VBT) on lower-limb strength performance and straight punch ability in collegiate boxers		
**Main Researcher**	Zhen Zhang		ID: 2003010003
**Degree/Title**	Doctor/Professor	School of Physical Education, Shanghai University of Sport, 650 Changhai Road.	Email: zhangzhen@sus.edu.cn<br>Tel: 13916012008
All personnel involved in human experiment research must study ethics-related laws and regulations before submitting an application.			Have you learned: YES☑ NO☐
**Co-investigator**	**Department**	ID	**Email**
Yemin Han	School of Physical Education and Sport Training	2321151092	2321151092@sus.edu.cn
**Project start time**	2025.2.29-2025.4.29		

Is there a project funding source for this study or has funding been applied for?

YES☐ NO☑

If the answer is yes, please provide the name of the project and the source of funding.

If has applied for funding, what is the status of the application?

☐Recently submitted ☑Have submitted, waiting for the result

☐Received funding

For financial support, please provide the funding source institution and approval number.
