# Supplementary material for: Effects of In-Season Velocity-Based Resistance Training on Boxing-Specific Performance in Collegiate Boxers: A Companion Report from a Randomized Controlled Trial
Source: J Funct Morphol Kinesiol. 2026 Aug 9;11(3):310. doi: 10.3390/jfmk11030310 (PMC13510493; doi:10.3390/jfmk11030310)
Supplement: Supplementary file 1 [file jfmk-11-00310-s001.zip › File S2. Informed Consent Form_Blank.docx.pdf]

Informed Consent Form

Study Title:

The Effect of In-Season Velocity-Based Resistance Training on Boxing-Specific Performance in Collegiate Boxers: A Randomized Controlled Trial

Research Institution:

School of Physical Education, Shanghai University of Sport

Principal Investigator:

Yemin Han

Email: 2321151092@sus.edu.cn

1. Background and Purpose

The purpose of this study is to compare the effects of velocity-based resistance training (VBT) and traditional percentage-based training (PBT) on boxing-specific performance in collegiate boxers. This research aims to provide scientific evidence to optimize strength and conditioning programs for combat sports athletes during the in-season period.

2. Study Procedures

A total of 28 male collegiate boxers will voluntarily participate in this study. Participants will be randomly assigned to one of two groups: a VBT group or a PBT group. The intervention will last for 8 weeks, with two supervised resistance training sessions per week.

Both groups will perform the same exercises, including the **back squat, Bulgarian**

split-squat, and deadlift, at 70% of their one-repetition maximum (1RM).

VBT Group: Training load and repetitions will be adjusted based on real-time velocity feedback using a linear position transducer. Each set will be terminated when the mean velocity decreases by more than 10% from the initial repetition.

PBT Group: Participants will perform a fixed number of repetitions (5 reps per set) with a constant load corresponding to 70% of 1RM.

Pre- and post-tests will include the following performance assessments:

Lead and rear straight punch (LSP and RSP) force measured using a boxing-specific dynamometer

Continuous punching frequency over 10 s, 30 s, and 1 min

3. Potential Risks and Discomforts

The study involves moderate-intensity resistance training under professional supervision. Participants may experience temporary muscle soreness or fatigue after training sessions. All sessions will be conducted under the guidance of qualified strength and conditioning coaches to minimize risk. If any discomfort or injury occurs, participation may be stopped immediately, and appropriate care will be provided.

4. Potential Benefits

Participants may experience improvements in punching power, endurance, and overall boxing performance. The results may also help optimize individualized training programs during the competitive season. Participants will receive feedback on their performance and guidance for safe resistance training practices.

5. Confidentiality

All personal information and collected data will be kept strictly confidential and will be used solely for research purposes. Participants will be assigned coded identifiers, and their names will not appear in any publication or presentation. Data will be securely stored and accessible only to the research team.

6. Voluntary Participation

Participation in this study is completely voluntary. You may refuse to participate or withdraw from the study at any time without penalty or loss of any training or academic benefits.

7. Contact Information

If you have any questions or concerns about this study, please contact:

Yemin Han

Shanghai University of Sport

Email: 2321151092@sus.edu.cn

8. Participant Acknowledgment

I have read and understood the information above. I have been informed about the study's purpose, procedures, potential risks, and benefits. I voluntarily agree to participate in this study.

Participant Name (Signature): ______________________Date: ____ / ____ / ____

Researcher Name (Signature): ______________________Date: ____ / ____ / ____
